# Surgical site infection after gastrointestinal surgery in children: an international, multicentre, prospective cohort study

**DOI:** 10.1136/bmjgh-2020-003429

**Published:** 2020-12-03

**Authors:** GlobalSurg Collaborative, Thomas M Drake

**Affiliations:** Department of Clinical Surgery, University of Edinburgh Division of Clinical and Surgical Sciences, Edinburgh, UK

**Keywords:** gastro-enterologic surgery, paediatrics

## Abstract

**Introduction:**

Surgical site infection (SSI) is one of the most common healthcare-associated infections (HAIs). However, there is a lack of data available about SSI in children worldwide, especially from low-income and middle-income countries. This study aimed to estimate the incidence of SSI in children and associations between SSI and morbidity across human development settings.

**Methods:**

A multicentre, international, prospective, validated cohort study of children aged under 16 years undergoing clean-contaminated, contaminated or dirty gastrointestinal surgery. Any hospital in the world providing paediatric surgery was eligible to contribute data between January and July 2016. The primary outcome was the incidence of SSI by 30 days. Relationships between explanatory variables and SSI were examined using multilevel logistic regression. Countries were stratified into high development, middle development and low development groups using the United Nations Human Development Index (HDI).

**Results:**

Of 1159 children across 181 hospitals in 51 countries, 523 (45·1%) children were from high HDI, 397 (34·2%) from middle HDI and 239 (20·6%) from low HDI countries. The 30-day SSI rate was 6.3% (33/523) in high HDI, 12·8% (51/397) in middle HDI and 24·7% (59/239) in low HDI countries. SSI was associated with higher incidence of 30-day mortality, intervention, organ-space infection and other HAIs, with the highest rates seen in low HDI countries. Median length of stay in patients who had an SSI was longer (7.0 days), compared with 3.0 days in patients who did not have an SSI. Use of laparoscopy was associated with significantly lower SSI rates, even after accounting for HDI.

**Conclusion:**

The odds of SSI in children is nearly four times greater in low HDI compared with high HDI countries. Policies to reduce SSI should be prioritised as part of the wider global agenda.

**Key questions**

**What is already known?**

- There is a lack of data describing the incidence and risk factors for surgical site infection (SSI) in children. This paucity of data is particularly prevalent in low-middle income populations (LMICs), who are likely to be affected most by SSI, yet no prospective, multicentric comparative data exist.

**What are the new findings?**

- Children undergoing gastrointestinal surgery in low-middle development countries are significantly more likely to have SSIs after surgery than their counterparts in high income settings. Use of laparoscopy was associated with lower odds of SSI.

**What do the new findings imply?**

- SSI is common in LMICs, occurring in up to a quarter of operations. Our data should be used as a foundation to inform clinical trials and to build initiatives to reduce this. SSI is associated with poorer outcomes and longer lengths of stay, which can be devastating for a family and for children’s well-being.

## Introduction

Surgical site infection (SSI) is one of the most common healthcare-associated infections (HAIs) following gastrointestinal surgery. In children, SSI has important social and economic consequences, leading to time away from school and lost working days by parents to assist children, putting family units at risk of catastrophic healthcare expenditure.^1 2^ Furthermore, the societal burdens of SSI in terms of healthcare costs and the requirements for antibiotic therapy have important consequences for affordability, antibiotic resistance and health system capacity.

Previous studies have predominately come from single hospitals, been heterogeneous in patient and procedure inclusion, and been inconsistent in the different diagnostic criteria for SSI.^3–5^ Furthermore, these studies are often limited in geographical scope, with one systematic review and meta-analysis of HAI identifying only three studies in paediatric patients in low-middle income countries.^6^ In these settings, SSI incidence is markedly higher than in high income countries.^7^

The primary aim of this study was to determine the worldwide SSI rate following gastrointestinal surgery in children while the secondary aim was to identify associations between SSI and morbidity among children by country development, according to the United Nation’s Human Development Index (HDI).

## Methods

### Patient public involvement

Patients and the public were involved in design and dissemination of this study, including representation from patients in low HDI countries. We included patients in the conception, design and steering of the overall GlobalSurg 2 Study and GlobalSurg collaborative.

### Study design

This international, multicentre, prospective cohort study was performed according to a published protocol and was registered on ClinicalTrials.gov (NCT02662231).^8^ Investigators were recruited via the international GlobalSurg collaborative research network, surgical associations, training colleges, social media and personal contacts. The structure of surgical collaborative research methodology has been described in detail previously.^9^ Briefly, small teams of local investigators collected data on prospectively determined items, coordinated by regional and national lead investigators, across short time windows, with pooled analysis by a central steering committee. A parallel analysis which also included adults (>16 years) has already been published elsewhere.^10^

This study is reported according to the STrengthening the Reporting of OBservational studies in Epidemiology guidelines and the Statistical Analyses and Methods in the Published Literature.^11 12^

Any hospital in the world performing gastrointestinal surgery on children was eligible to participate in this study. Hospitals could be secondary or tertiary healthcare facilities. Our network includes both small community hospitals and large tertiary referral hospitals. There was no minimum case-volume, or centre-specific requirements to take part. Participating clinicians registered online and had to successfully pass a mandatory online training module to standardise data collection. Investigators included consecutive patients over a 2-week period selected during the course of the study. Multiple non-overlapping 2-week study periods were encouraged. Children (<16 years) undergoing elective or emergency gastrointestinal resection were eligible for inclusion. Gastrointestinal resection was defined as complete transection and removal of a segment of the gastrointestinal tract at any point from oesophagus to rectum, including appendix and gall bladder as well as reversal or formation of a stoma.

Data were collected on patient-level risk factors, which included preoperative variables (age, gender, admission to procedure time, American Society of Anesthesiologists (ASA) grade, urgency of operation, presence of malaria or HIV), disease variables (pathology) and operative variables (urgency, procedure start time, operative approach, WHO surgical safety checklist use, antibiotic prophylaxis and intraoperative contamination). Intraoperative contamination was measured by the operating surgeon and defined as follows; clean-contaminated (an incision through the respiratory, alimentary or genitourinary tract under controlled conditions with no direct contamination encountered), contaminated (an operation where there is major break in sterile technique or gross spillage from the gastrointestinal tract, or an incision where acute non-purulent inflammation is encountered or where procedures involve traumatic wounds that have been open for between 12 hours and 24 hours) and dirty (an incision undertaken where viscera are perforated, where acute inflammation or necrosis is encountered, or where there is delayed operation on traumatic wounds) procedures.

Within each team, quality assurance was guaranteed by at least one consultant or attending-level surgeon. Data were recorded prospectively and stored on a secure, internet-based, user-encrypted platform (REDCap).^13^

### Primary outcome

The primary outcome measure was the 30-day postoperative SSI rate according to the Centre for Disease Control and Prevention criteria for SSI.^14^ SSI rates were measured at 30 days following surgery either in person or by computer record/chart review. When 30-day follow-up was not possible, SSI was measured at the point of discharge. Surgical teams were encouraged to assess patients at 30 days (either in-person or via telephone). Secondary outcome measures included the 30-day postoperative mortality rate, reintervention rate, rate of wound organ-space inefction, rate of other HAIs and length of stay. Data were also collected on antimicrobial therapy and microbiology culture from patients with SSI. Antibiotic resistance was defined as resistance in the species which was cultured to the antibiotic administered for prophylaxis.

### Statistical analysis

As described in the protocol, countries were stratified into tertiles of development using the United Nations HDI. This metric is calculated from a composite of country-level statistics including life expectancy, income and education (http://hdr.undp.org/en/statistics). Variation across HDI tertiles were described first using simple summary statistics, including the number of patients and proportions. Continuous variables with normal distributions were summarised using the mean and SD, otherwise the median and IQR was used. Differences were tested using Pearson’s χ^2^ test for categorical variables or the Kruskall-Wallis test for continuous variables. Multilevel logistic regression models were used to adjust for patient-level (fixed effects) and centre-level (random effects) variations to identify factors which were independently associated with the study outcomes. Effects estimates from these models are presented as ORs alongside the corresponding 95% CIs. Clinically plausible and relevant variables were entered into the models, and interactions explored. Model selection was guided through minimisation of the Akaike information criterion (AIC). Statistical significance was taken at the level of p≤0·05, as specified a priori. All analysis was performed in R V. 3.4.4 (R Foundation for Statistical Computing, Vienna, Austria) using the tidyverse, lme4 and finalfit packages. As part of this project, an interactive data set explorer is available online at http://ssi.globalsurg.org/.

## Results

**Figure 1:**
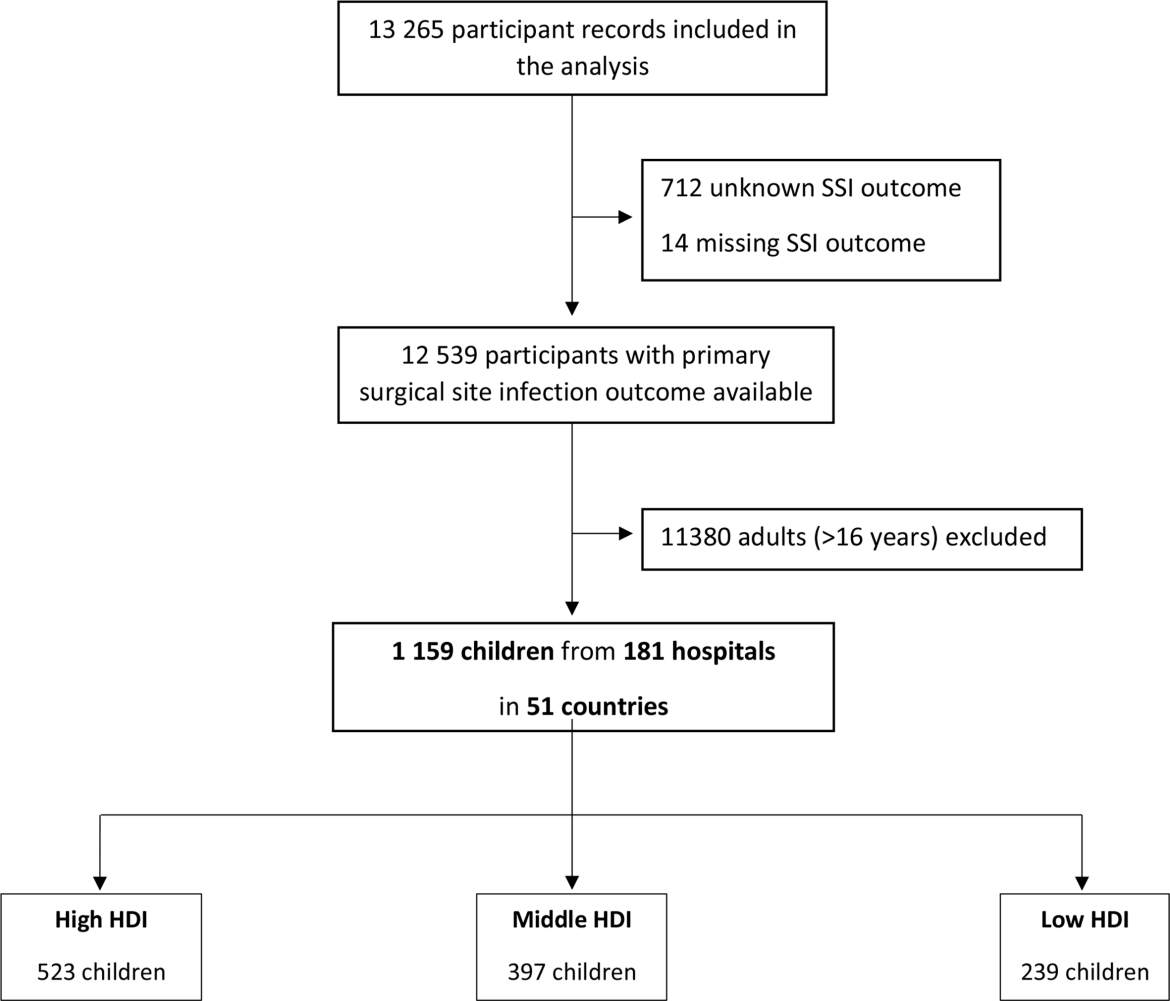
Participant inclusion flow chart. HDI, Human Development Index; SSI, surgical site infection.

Overall 1159 children across 181 hospitals were included from 51 countries during the study period. Of these, 523 (45·1%) children were from high HDI countries, 397 (34·2%) from middle HDI countries and 239 (20·6%) from low HDI countries. The overall study inclusion flow chart for the GlobalSurg 2 Study and this paediatric subset is shown in figure 1. There were minimal missing data. Validation of the overall GlobalSurg 2 data set from a sample of 1476 patients found a 93% correct case ascertainment rate. Data accuracy was high, with Cohen’s κ >0·90 and Pearson’s correlation coefficient 0·99, when data entered at primary entry and validation were compared.

**Figure 2:**
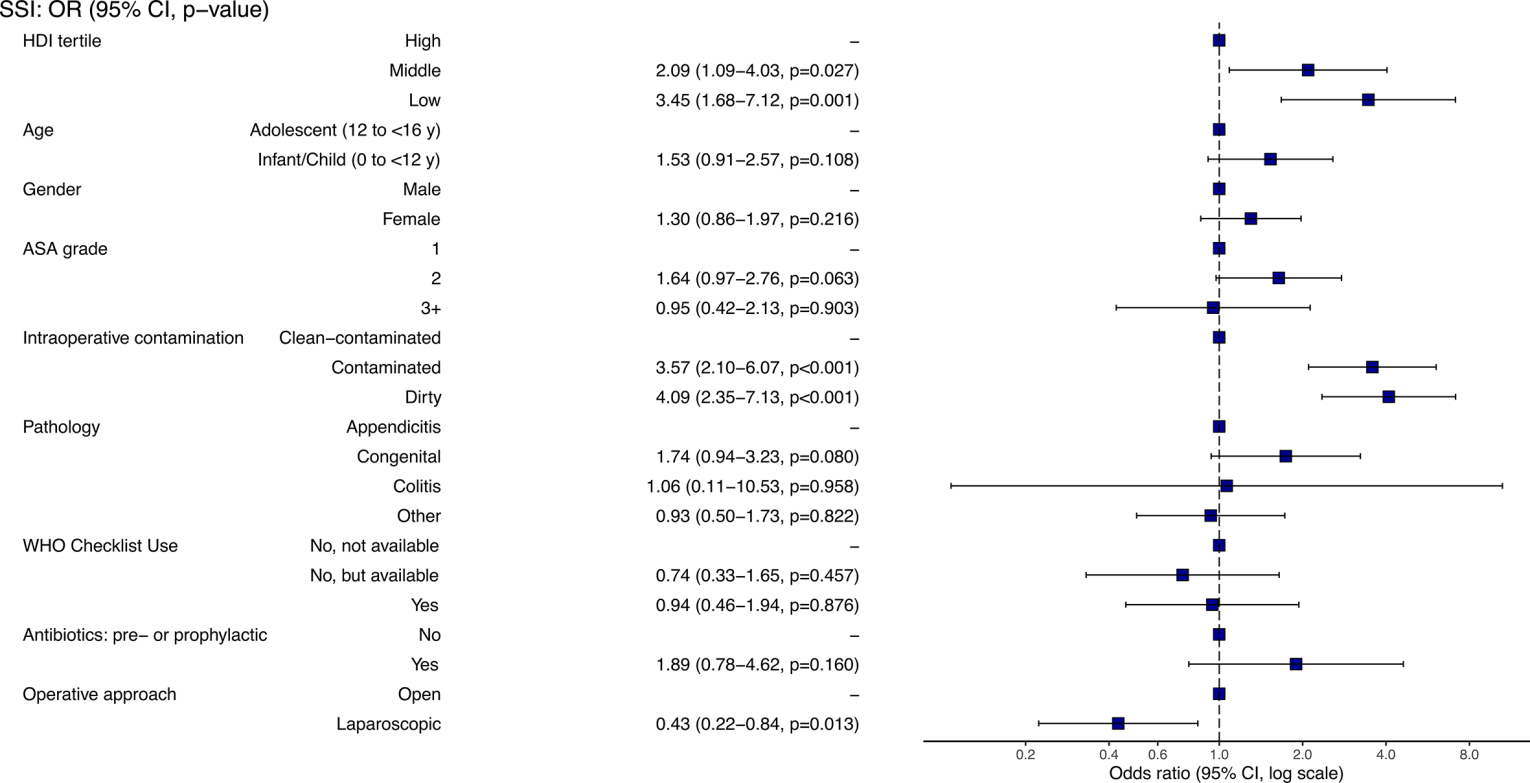
Multilevel, multivariable models for 30-day surgical site infection (SSI) in patients undergoing non-appendicitis surgery (A) or surgery for appendicitis (B). ASA, American Society of Anesthesiologists.

In low HDI settings, patients were younger, with the rate of neonatal surgery fivefold and threefold higher than in high HDI and middle HDI settings, respectively (table 1). Patients in low HDI settings had a preponderance towards being male, with higher ASA grades and were more likely to undergo elective surgery than patients in high HDI settings. Use of laparoscopic surgery was significantly higher in the high HDI group, with surgery more likely to be performed closer to the time of admission. The WHO surgical safety checklist was used in 86·0% (450/523) of patients in high HDI, 43·8% (174/397) of patients in middle HDI and 45·2% (81/239) of patients in low HDI settings. The rate of antibiotic prophylaxis was significantly lower in high HDI settings, despite a higher proportion of dirty procedures performed. Contaminated surgery was most commonly performed in low HDI countries and clean-contaminated procedures in middle HDI countries. Acute appendicitis was the most common indication for surgery across all HDI groups, comprising 70·8% (820/1159) of all cases (online supplemental table S1 and S2).

10.1136/bmjgh-2020-003429.supp1 Supplementary data

**Table 1:** Patient characteristics by HDI

	High HDI (n=523)	Middle HDI (n=397)	Low HDI (n=239)	P value
Age				
Adolescent (12 years to <16 years)	207 (39.6)	157 (39.5)	72 (30.1)	<0.001
Child (2 years to <12 years)	274 (52.4)	172 (43.3)	93 (38.9)	
Infant (1 month to <2 years)	28 (5.4)	46 (11.6)	38 (15.9)	
Neonate (<1 month)	14 (2.7)	22 (5.5)	36 (15.1)	
Gender				
Male	307 (58.7)	212 (53.4)	151 (63.2)	0.002
Female	210 (40.2)	166 (41.8)	84 (35.1)	
Missing	6 (1.1)	19 (4.8)	4 (1.7)	
ASA grade				
1	429 (82.0)	314 (79.1)	128 (53.6)	<0.001
2	66 (12.6)	41 (10.3)	73 (30.5)	
3+	19 (3.6)	13 (3.3)	34 (14.2)	
Missing	9 (1.7)	29 (7.3)	4 (1.7)	
Urgency				
Elective	59 (11.3)	79 (19.9)	65 (27.2)	<0.001
Emergency	463 (88.5)	318 (80.1)	174 (72.8)	
Missing	1 (0.2)	0 (0.0)	0 (0.0)	
Operative approach				
Open	235 (44.9)	330 (83.1)	231 (96.7)	<0.001
Laparoscopic	288 (55.1)	67 (16.9)	8 (3.3)	
Procedure start time				
08:00–18:00	331 (63.3)	222 (55.9)	155 (64.9)	0.002
18:00–22:00	106 (20.3)	73 (18.4)	49 (20.5)	
22:00–08:00	86 (16.4)	102 (25.7)	35 (14.6)	
Admission to procedure time (hours)				
<6	140 (26.8)	189 (47.6)	43 (18.0)	<0.001
6–11	94 (18.0)	49 (12.3)	33 (13.8)	
12–23	157 (30.0)	60 (15.1)	45 (18.8)	
24–47	76 (14.5)	30 (7.6)	48 (20.1)	
48+	37 (7.1)	39 (9.8)	57 (23.8)	
Missing	19 (3.6)	30 (7.6)	13 (5.4)	
WHO checklist use				
No, not available	43 (8.2)	146 (36.8)	44 (18.4)	<0.001
No, but available	20 (3.8)	74 (18.6)	81 (33.9)	
Yes	450 (86.0)	174 (43.8)	108 (45.2)	
Missing	10 (1.9)	3 (0.8)	6 (2.5)	
Antibiotics: preoperative or prophylactic				
No	80 (15.3)	59 (14.9)	7 (2.9)	<0.001
Yes	438 (83.7)	333 (83.9)	230 (96.2)	
Missing	5 (1.0)	5 (1.3)	2 (0.8)	
Pathology				
Appendicitis	438 (83.7)	281 (70.8)	101 (42.3)	<0.001
Congenital	36 (6.9)	43 (10.8)	79 (33.1)	
Colitis	5 (1.0)	1 (0.3)	2 (0.8)	
Other	44 (8.4)	72 (18.1)	57 (23.8)	
Intraoperative contamination				
Clean-contaminated	347 (66.3)	314 (79.1)	157 (65.7)	<0.001
Contaminated	63 (12.0)	45 (11.3)	51 (21.3)	
Dirty	109 (20.8)	36 (9.1)	31 (13.0)	
Missing	4 (0.8)	2 (0.5)	0 (0.0)	

Data are presented as n (%). Statistical tests are χ^2^.

ASA, American Society of Anesthesiologists; HDI, Human Development Index.

The 30-day SSI rate overall was 12·3% (143/1159). Rates varied by HDI, with an infection rate of 6·3% (33/523), 12·8% (51/397) and 24·7% (59/239) in high HDI, middle HDI and low HDI countries, respectively (tables 2 and 3). In high HDI settings, SSI was diagnosed at similar rates in-hospital and after discharge (before discharge 2.9% (15/523), after discharge 3.4% (18/523)). In middle HDI and low HDI countries, SSI was diagnosed in-hospital more frequently (middle HDI before discharge 6.8% (27/397), middle HDI after discharge 6.0% (24/397), low HDI before discharge 18.4% (44/239), low HDI after discharge 6.3% (15/239)).

**Table 2:** SSI by age and HDI

HDI tertile	Age group	No SSI (n=1016)	SSI present (n=143)
High	Adolescent (12 years to <16 years	199 (96.1)	8 (3.9)
	Child (2 years to <12 years)	254 (92.7)	20 (7.3)
	Infant (1 month to <2 years)	26 (92.9)	2 (7.1)
	Neonate (<1 month)	11 (78.6)	3 (21.4)
Middle	Adolescent (12 years to <16 years)	139 (88.5)	18 (11.5)
	Child (2 years to <12 years)	145 (84.3)	27 (15.7)
	Infant (1 month to <2 years)	42 (91.3)	4 (8.7)
	Neonate (<1 month)	20 (90.9)	2 (9.1)
Low	Adolescent (12 years to <16 years)	64 (88.9)	8 (11.1)
	Child (2 years to <12 years)	64 (68.8)	29 (31.2)
	Infant (1 month to <2 years)	27 (71.1)	11 (28.9)
	Neonate (<1 month)	25 (69.4)	11 (30.6)

Data are presented as n (%)

HDI, Human Development Index; SSI, Surgical Site Infection.

**Table 3:** Outcomes by SSI

	No SSI (n=1016)	SSI present (n=143)	P value
30-day mortality			
Alive	971 (95.6)	132 (92.3)	0.006
Dead	20 (2.0)	9 (6.3)	
Missing	25 (2.5)	2 (1.4)	
30-day reintervention			
No	990 (97.4)	107 (74.8)	<0.001
Yes	17 (1.7)	33 (23.1)	
Missing	9 (0.9)	3 (2.1)	
30-day organ-space infection			
No	992 (97.6)	113 (79.0)	<0.001
Yes	17 (1.7)	26 (18.2)	
Missing	7 (0.7)	4 (2.8)	
Other healthcare-associated infection (HAI)			
No	984 (96.9)	121 (84.6)	<0.001
Yes	24 (2.4)	18 (12.6)	
Missing	8 (0.8)	4 (2.8)	
Length of stay (by SSI)			
Median days (IQR)	3.0 (5.0)	7.0 (8.0)	<0.001*

Data are presented as n (%). Statistical tests are χ^2^, besides where the Kruskall-Wallis test was used as denoted by *.

SSI, surgical site infection.

**Figure 3:**
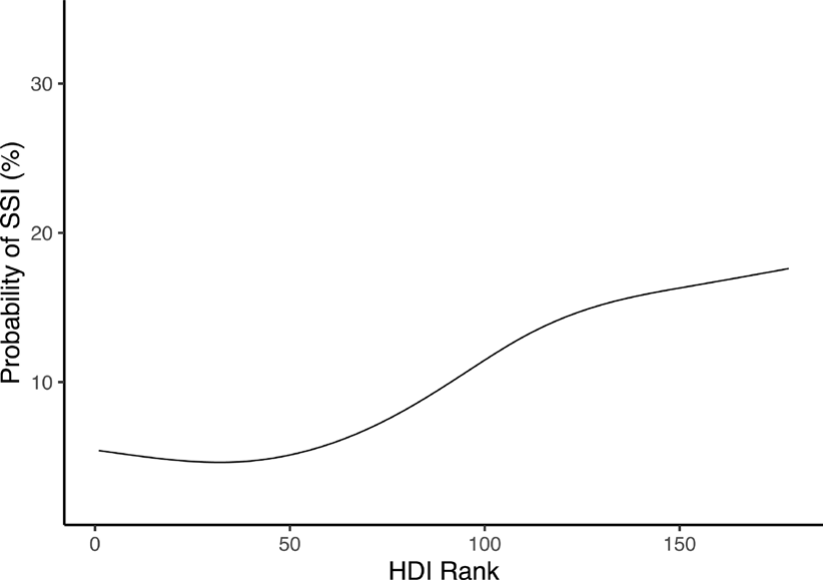
Relationship between HDI rank and probability of SSI (with rank 1 being most highly developed). adjusted for patient-level risk factors including age, sex, use of who checklist, antibiotic prophylaxis, intraoperative contamination and use of laparoscopy. HDI, Human Development Index; SSI, surgical site infection.

SSI was significantly more common in contaminated surgery compared with clean-contaminated surgery across all HDI settings (11·6% vs 3·5% in high, 25·9% vs 9·2% in middle and 39·0% vs 17·2% in low), (online supplemental table S3). Similarly, rates of organ-space infection and HAIs were higher in low HDI settings (table 4). The 30-day mortality and reintervention rates were also significantly higher in patients from low HDI countries when compared with those in middle HDI and high HDI groups. In univariable analyses, low HDI was associated with an increased odds of SSI (onine supplemental table S4). Patient-level factors which were also associated with SSI included non-use of the WHO checklist, the degree of intraoperative contamination and the use of open surgery. When adjusted for clinically plausible explanatory variables, low HDI, intraoperative contamination and open surgery remained significantly associated with higher odds of SSI (figure 2, online supplemental table S4). When HDI was treated as continuous, SSI was correlated with lower country development (figure 3, rank 1 being most developed).

**Table 4:** Outcomes by HDI

	High HDI (n=523)	Middle HDI (n=397)	Low HDI (n=239)	P value
SSI				
No	490 (93.7)	346 (87.2)	180 (75.3)	<0.001
Yes	33 (6.3)	51 (12.8)	59 (24.7)	
30-day mortality				
Alive	504 (96.4)	379 (95.5)	220 (92.1)	<0.001
Dead	1 (0.2)	13 (3.3)	15 (6.3)	
Missing	18 (3.4)	5 (1.3)	4 (1.7)	
30-day reintervention				
No	503 (96.2)	379 (95.5)	215 (90.0)	0.001
Yes	18 (3.4)	11 (2.8)	21 (8.8)	
Missing	2 (0.4)	7 (1.8)	3 (1.3)	
30-day organ-space inefction				
No	502 (96.0)	382 (96.2)	221 (92.5)	0.027
Yes	16 (3.1)	10 (2.5)	17 (7.1)	
Missing	5 (1.0)	5 (1.3)	1 (0.4)	
Healthcare-associated infection (HAI)				
No	508 (97.1)	381 (96.0)	216 (90.4)	<0.001
Yes	12 (2.3)	10 (2.5)	20 (8.4)	
Missing	3 (0.6)	6 (1.5)	3 (1.3)	
Length of stay (by HDI)				
Median days (IQR)	3.0 (4.0)	3.0 (5.0)	6.0 (6.0)	<0.001*

Data are presented as n (%). Statistical tests are χ^2^, besides where the Kruskall-Wallis test was used as denoted by *.

HDI, Human Development Index; SSI, surgical site infection.

### Organisms and antibiotic resistance

In the 12·3% (143/1159) of patients who developed an SSI, 94·4% (135/143) had access to microbiological testing and of these 36·3% (49/135) had organisms cultured from their wound. The most common organisms were coliforms, present in 46·9% of culture-positive patients (23/49, online supplemental table S5) followed by *Staphylococcus aureus* species present in 22·4% of culture-positive patients (11/49, online supplemental table S4). Of those with sensitivity testing performed (35/49), 28·6% (10/35) patients were found to have organisms resistant to antibiotic prophylaxis.

## Discussion

This patient-level, prospective, validated global study found that children in low HDI and middle HDI countries were significantly more likely to suffer from SSI than their counterparts in high HDI countries. In children who did suffer an SSI, there was an association with further complications, including reintervention, and a longer length of stay. Furthermore, we found that children in low-middle income countries had a far higher in-hospital rate of SSI, when compared with children in middle-development and high-development settings.

The main finding of higher incidence and poorer outcomes associated with SSIs in children in low HDI settings likely reflects a complex interplay of potential factors, including weaker preventative measures such as sanitation and vaccination, nutritional issues, shortcomings in knowledge and application of basic infection control, understaffing and overcrowding in medical facilities, absence of local and national guidelines and policies, and the wider lack of structural and financial enablers to improve access to high-quality, affordable healthcare.^15 16^ These inequalities are likely to contribute towards healthcare environments which predispose patients to HAIs, including SSI, which are known contributors to mortality. The contributory inequalities at the point of care have already been well documented in low-middle income countries, including a lack of timely access leading to delays in presentation and more advanced disease, and a lack of capacity in surgery and perioperative care compounding the risks of delayed presentation. Lack of postoperative care, including appropriate intensive care facilities, may also contribute to poorer outcomes. Shortages in the staff and facilities to perform routine microbiological testing may result in ineffectual prophylactic antibiotic usage.^17^ We found use of laparoscopy was associated with lower SSI rates, an effect which persisted when HDI was accounted for. Barriers to uptake of surgical technologies (including training, treatment costs and lack of supportive infrastructure for technologies including servicing, equipment support staff, distribution and repair capability), such as laparoscopy, are likely to greatly affect LMICs and increase the observed rate of SSI in children.

The prospective, multicentric design of this research is a major strength, drawing from a large and diverse international patient population. Many global health studies which compare international practice rely on estimates derived from historical data or statistical modelling. Our patient-level approach gives us the ability to look more closely at where SSI is occurring, in which patients, and to identify individuals at the highest risk with far greater power than modelling studies. To explore the effect of HDI on SSI, we constructed risk-adjusted multilevel models which adjusted for patient-level and centre-level effects. In contrast, previous patient-level studies have suffered from low numbers of patients and use heterogeneous diagnostic criteria for SSI. This may explain why we found higher rates of SSI than previous studies. Nonetheless, there are several limitations inherent to our study design, including the inability to follow-up every patient 30 days after surgery. Taking a pragmatic approach given the dispersed global nature of this study, our methodology did not standardise microbiological specimen collection, laboratory assessment, techniques or definitions. We also did not collect centre-level data on the microbiological testing facilities available at each centre. This instead relied on local protocols, including for determining antimicrobial resistance, potentially introducing variation. Although we undertook data validation, there is still the potential for missed cases or inaccurate data. However, the large number of patients, a prospective protocol combined with compulsory investigator training, and the use of local coordinators will have helped minimise any potential bias. A further limitation may be that surgery in children is a highly centralised specialty. In our study, we may have not been able to fully capture the casemix of surgeons in rural settings owing to the requirement for internet to contribute to this study. In high-income countries, smaller centres tend to perform only appendicectomy on children and more complicated surgical cases are referred to a tertiary unit, which may limit the generalisability of our findings.

There is good evidence to support surgical technologies that reduce SSI in abdominal surgery. We identified a significantly lower use of laparoscopic surgery in low-income and middle-income countries. While this might point to a lack of training, or familiarity with minimal access procedures combined with higher treatment costs, laparoscopic surgery remains one such technology known to reduce SSI.^18^ The most common procedure across all settings was appendicectomy, which can be readily performed using the laparoscopic approach. Our data support that the laparoscopic approach is associated with significantly lower rates of SSI, independent of HDI setting. Given the higher rates of SSI in low-income settings, the absolute benefit of laparoscopy is likely to be even greater than the effect observed in high-income countries. Our previous analysis of both children and adults undergoing surgery suggested a number needed to treat, with laparoscopic rather than open surgery, of 6 to prevent one SSI in low HDI countries managing perforated appendicitis.^19^ Therefore, if a low-cost and durable system could be developed to enable greater adoption of laparoscopic surgery for appendicectomy in low HDI settings, this could provide a straightforward means of rapidly reducing SSI through deployment of surgical technologies.^20^

However, propagation of technologies to lower-resource settings will not fully address the underlying, disease-specific and casemix-related reasons for higher SSI rates. In this study, there was a significantly higher proportion of infants and neonates in the low HDI and middle HDI countries, with a significantly higher proportion of neonates undergoing abdominal surgery in low HDI countries. This may reflect the unequal burden of disease in these settings, with a much larger volume of congenital malformations, trauma and other injuries, and infectious causes in low HDI settings, contrasting with high HDI settings, where a wider range of procedures are performed. These are complex procedures, where contamination is frequently encountered, which is reflected in our data. These problems are unlikely to go away soon, as there is a large unmet need and burden of paediatric disease that requires surgery. An estimated 94% of congenital anomalies occur in low HDI countries, where, due to sociocultural, economic and structural factors, access to high-quality paediatric surgery may be limited.^21 22^ The global burden of childhood trauma and injury, including road traffic collisions, falls, burns, drowning and poisoning, disproportionately affects low-middle income countries (LMICs), and progress addressing this has been slow.^23^ The distribution and incidence of numerous surgically relevant infectious diseases also disproportionately affects LMICs.^24^ Therefore ensuring paediatric surgery in these settings is safe and equitable, and must be made a global health priority.

Given that children make up approximately 50% of the population in LMICs, ensuring access to safe surgery with good outcomes is vital. However, data describing outcomes following paediatric surgery are lacking in many countries, representing a major research gap and an obstacle to delivering safe surgery with good clinical outcomes. Our data show that paediatric SSI rate in low development and middle development countries is disproportionately higher than in high development settings. As a result, the expected expansion of surgical services in LMICs could have unintended consequences if this disparity in outcomes is not simultaneously addressed.^25^ At the population level, preventing SSI after surgery is also an important component in the global fight against antimicrobial resistance. Several of the WHO recommendations for SSI prevention rely on non-antibiotic mechanisms (eg, preoperative bathing and surgical site sterilisation). Ensuring effective implementation of these may provide a useful way of reducing reliance on antibiotics and consequences such as antimicrobial resistance, however, research on interventions to curb the occurrence of SSI in lower-resource settings is currently extremely limited.^26 27^ Future research should aim to quantify the affects of SSI on long-term outcomes in children, particularly the consequences for children in LMICs. Understanding the impact of SSI in these settings on families, educational attainment and developmental outcomes is important to assessing the true burden that these infections have. Furthermore, clinical trials should be undertaken to identify implementable methods of reducing SSI in children across the world, which capture these longer-term outcomes.

## Conclusions

Worldwide, SSI represents a significant burden of postoperative morbidity in children who receive surgery. Focused initiatives and research, aiming to reduce SSIs in children should be a key priority for global surgery agendas.
