# Supplementary material for: Surgical site infection after gastrointestinal surgery in children: an international, multicentre, prospective cohort study
Source: BMJ Glob Health. 2020 Dec 3;5(12):e003429. doi: 10.1136/bmjgh-2020-003429 (PMC7716674; doi:10.1136/bmjgh-2020-003429)
Supplement: Supplementary data [file bmjgh-2020-003429supp001.pdf]

Supplementary Tables

Table S1 – Pathology by HDI

	High HDI (n = 523)	Middle HDI (n = 397)	Low HDI (n = 239)	Total (n = 1159)
K35 Appendicitis	438 (83·7)	281 (70·8)	101 (42·3)	820 (70·8)
Q43 Congenital: Other	26 (5·0)	32 (8·1)	65 (27·2)	123 (10·6)
K561 Intestinal obstruction: Intussusception	6 (1·1)	11 (2·8)	17 (7·1)	34 (2·9)
K80 Cholelithiasis / cholecystitis (gallstones)	14 (2·7)	11 (2·8)	1 (0·4)	26 (2·2)
Q431 Hirschsprung's disease	2 (0·4)	5 (1·3)	9 (3·8)	16 (1·4)
D13 Neoplasm: any benign	1 (0·2)	9 (2·3)	4 (1·7)	14 (1·2)
00 No disease identified	1 (0·2)	10 (2·5)	0 (0·0)	11 (0·9)
Q430 Congenital: Meckel diverticulum	6 (1·1)	2 (0·5)	3 (1·3)	11 (0·9)
K562 Intestinal obstruction: Volvulus	2 (0·4)	3 (0·8)	4 (1·7)	9 (0·8)
A01 Infection, Typhoid / paratyphoid	1 (0·2)	0 (0·0)	7 (2·9)	8 (0·7)
K565 Intestinal obstruction: Adhesions	4 (0·8)	2 (0·5)	2 (0·8)	8 (0·7)
Q793 Congenital: Gastroschisis	2 (0·4)	4 (1·0)	2 (0·8)	8 (0·7)
Y83 Complication of previous surgical operation / procedure	2 (0·4)	0 (0·0)	6 (2·5)	8 (0·7)
K52 Colitis/gastroenteritis: Other noninfective inc· ischemic bowel	2 (0·4)	4 (1·0)	1 (0·4)	7 (0·6)
C26 Neoplasm: any malignant (cancer)	1 (0·2)	3 (0·8)	2 (0·8)	6 (0·5)
K46 Hernia: any abdominal hernia	1 (0·2)	2 (0·5)	2 (0·8)	5 (0·4)
K51 Colitis/gastroenteritis: Ulcerative colitis	4 (0·8)	1 (0·3)	0 (0·0)	5 (0·4)
S31 Trauma: penetrating	0 (0·0)	3 (0·8)	2 (0·8)	5 (0·4)
99 Other diagnosis (please specify; please try to avoid using)	2 (0·4)	0 (0·0)	2 (0·8)	4 (0·3)
A49 Infection: other	0 (0·0)	1 (0·3)	3 (1·3)	4 (0·3)
K50 Colitis/gastroenteritis: Crohns disease	3 (0·6)	1 (0·3)	0 (0·0)	4 (0·3)
S38 Trauma: non-penetrating / blunt	0 (0·0)	2 (0·5)	2 (0·8)	4 (0·3)
A09 Infection, Other infectious gastroenteritis / colitis	1 (0·2)	0 (0·0)	2 (0·8)	3 (0·3)
K55 Bleeding: small bowel / colon with no malignancy	0 (0·0)	3 (0·8)	0 (0·0)	3 (0·3)
K85 Acute pancreatitis	3 (0·6)	0 (0·0)	0 (0·0)	3 (0·3)
E66 Obesity	0 (0·0)	2 (0·5)	0 (0·0)	2 (0·2)
K275 Peptic ulcer: perforation	1 (0·2)	1 (0·3)	0 (0·0)	2 (0·2)
T54 Toxic effect of corrosive substances	0 (0·0)	0 (0·0)	2 (0·8)	2 (0·2)
K631 Perforation of intestine	0 (0·0)	1 (0·3)	0 (0·0)	1 (0·1)
K631 Stercoral perforation of colon	0 (0·0)	1 (0·3)	0 (0·0)	1 (0·1)
K831 Perforation of bile duct	0 (0·0)	1 (0·3)	0 (0·0)	1 (0·1)
Missing	0 (0·0)	1 (0·3)	0 (0·0)	1 (0·1)

Pathology is categorized into ICD10 codes. Number of patients undergoing a surgery for a specific pathology is presented as number of patients (%).

. *BMJ Global Health* 2020; 5:e003429. doi: 10.1136/bmjgh-2020-003429

Table S2 – Procedures by HDI

	High HDI (n = 523)	Middle HDI (n = 397)	Low HDI (n = 239)	Total (n = 1159)
H01 Appendix: Emergency excision of appendix	442 (84·5)	286 (72·0)	104 (43·5)	832 (71·8)
G58 Small bowel: Excision of small bowel	21 (4·0)	16 (4·0)	21 (8·8)	58 (5·0)
H15 Colon: Formation of any colonic stoma	7 (1·3)	7 (1·8)	23 (9·6)	37 (3·2)
G67 Small bowel: Other open operations on small bowel	9 (1·7)	17 (4·3)	10 (4·2)	36 (3·1)
H46 Rectum: Other open operations on rectum	2 (0·4)	8 (2·0)	24 (10·0)	34 (2·9)
J18 Gallbladder: Excision of gall bladder	16 (3·1)	12 (3·0)	2 (0·8)	30 (2·6)
H19 Colon: Other open operations on colon	4 (0·8)	7 (1·8)	13 (5·4)	24 (2·1)
H11 Colon: Other excision of colon	2 (0·4)	13 (3·3)	5 (2·1)	20 (1·7)
H07 Colon: Excision of right hemicolon	2 (0·4)	4 (1·0)	8 (3·3)	14 (1·2)
G70 Small bowel: Excision of Meckels diverticulum	5 (1·0)	3 (0·8)	3 (1·3)	11 (0·9)
H10 Colon: Excision of sigmoid colon	2 (0·4)	5 (1·3)	2 (0·8)	9 (0·8)
T281 Abdomen: Closure of gastroschisis/exomphalos	2 (0·4)	5 (1·3)	2 (0·8)	9 (0·8)
G74 Small bowel: Formation of ileostomy	0 (0·0)	2 (0·5)	5 (2·1)	7 (0·6)
G536 Duodenum: Correction of malrotation	1 (0·2)	0 (0·0)	5 (2·1)	6 (0·5)
H09 Colon: Excision of left hemicolon	2 (0·4)	2 (0·5)	2 (0·8)	6 (0·5)
H33 Rectum: Excision of rectum	2 (0·4)	1 (0·3)	3 (1·3)	6 (0·5)
G28 Stomach: Partial excision of stomach	0 (0·0)	4 (1·0)	1 (0·4)	5 (0·4)
H05 Colon: Total excision of colon	1 (0·2)	3 (0·8)	1 (0·4)	5 (0·4)
H06 Colon: Extended excision of right hemicolon	1 (0·2)	0 (0·0)	4 (1·7)	5 (0·4)
H04 Colon: Total excision of colon and rectum	2 (0·4)	1 (0·3)	0 (0·0)	3 (0·3)
G32 Stomach: Connection of stomach to jejunum	0 (0·0)	0 (0·0)	1 (0·4)	1 (0·1)
J23 Gallbladder: Other open operations on gall bladder	0 (0·0)	1 (0·3)	0 (0·0)	1 (0·1)

Procedures are categorized into OPCS4 codes. Number of patients undergoing a specific procedure is presented as number of patients (%).

Table S3 - SSI by contamination and HDI

HDI tertile	Intraoperative contamination	No SSI (n = 1016)	SSI Present (n = 143)
High	Clean-contaminated	335 (96·5)	12 (3·5)
	Contaminated/Dirty	152 (88·4)	20 (11·6)
	Unknown	3 (75·0)	1 (25·0)
Middle	Clean-contaminated	285 (90·8)	29 (9·2)
	Contaminated/Dirty	60 (74·1)	21 (25·9)
	Unknown	1 (50·0)	1 (50·0)
Low	Clean-contaminated	130 (82·8)	27 (17·2)
	Contaminated/Dirty	50 (61·0)	32 (39·0)
	Unknown	0 (0·0)	0 (0·0)

HDI – Human Development Index, SSI – Surgical Site Infection. Data are presented as n (%).

Table S4 - SSI Model

Dependent: SSI		No	Yes	OR (univariable)	OR (multilevel)
HDI tertile	High	490 (93.7)	33 (6.3)	-	-
	Middle	346 (87.2)	51 (12.8)	2.19 (1.39-3.49, p=0.001)	2.09 (1.09-4.03, p=0.027)
	Low	180 (75.3)	59 (24.7)	4.87 (3.10-7.77, p<0.001)	3.45 (1.68-7.12, p=0.001)
Age	Adolescent (12 to <16 y)	402 (92.2)	34 (7.8)	-	-
	Infant/Child (0 to <12 y)	614 (84.9)	109 (15.1)	2.10 (1.41-3.19, p<0.001)	1.53 (0.91-2.57, p=0.108)
Gender	Male	591 (88.2)	79 (11.8)	-	-
	Female	397 (86.3)	63 (13.7)	1.19 (0.83-1.69, p=0.343)	1.30 (0.86-1.97, p=0.216)
ASA grade	1	786 (90.2)	85 (9.8)	-	-
	2	138 (76.7)	42 (23.3)	2.81 (1.85-4.23, p<0.001)	1.64 (0.97-2.76, p=0.063)
	3+	54 (81.8)	12 (18.2)	2.05 (1.01-3.87, p=0.034)	0.95 (0.42-2.13, p=0.903)
Intraoperative contamination	Clean-contaminated	750 (91.7)	68 (8.3)	-	-
	Contaminated	121 (76.1)	38 (23.9)	3.46 (2.21-5.36, p<0.001)	3.57 (2.10-6.07, p<0.001)
	Dirty	141 (80.1)	35 (19.9)	2.74 (1.74-4.25, p<0.001)	4.09 (2.35-7.13, p<0.001)
Pathology	Appendicitis	746 (91.0)	74 (9.0)	-	-
	Congenital	116 (73.4)	42 (26.6)	3.65 (2.37-5.57, p<0.001)	1.74 (0.94-3.23, p=0.080)
	Colitis	7 (87.5)	1 (12.5)	1.44 (0.08-8.25, p=0.735)	1.06 (0.11-10.53, p=0.958)
	Other	147 (85.0)	26 (15.0)	1.78 (1.09-2.85, p=0.018)	0.93 (0.50-1.73, p=0.822)
WHO Checklist Use	No, not available	196 (84.1)	37 (15.9)	-	-
	No, but available	147 (84.0)	28 (16.0)	1.01 (0.59-1.72, p=0.974)	0.74 (0.33-1.65, p=0.457)
	Yes	654 (89.3)	78 (10.7)	0.63 (0.42-0.97, p=0.033)	0.94 (0.46-1.94, p=0.876)
Antibiotics: pre- or prophylactic	No	138 (94.5)	8 (5.5)	-	-
	Yes	869 (86.8)	132 (13.2)	2.62 (1.34-5.93, p=0.010)	1.89 (0.78-4.62, p=0.160)
Operative approach	Open	669 (84.0)	127 (16.0)	-	-

Laparoscopic	347 (95.6)	16 (4.4)	0.24 (0.14-0.40, p<0.001)	0.43 (0.22-0.84, p=0.013)

Number in model = 1061, Number of groups = 172, AIC = 706.8, C-statistic = 0.825

HDI – Human Development Index, OR – Odds Ratio, ASA – American Society of Anesthesiologists', y – Years, WHO – World Health Organisation. Data are presented as n (%) and odds ratios (OR) alongside the corresponding (95% confidence interval).

. *BMJ Global Health* 2020; 5:e003429. doi: 10.1136/bmjgh-2020-003429

Table S5 - Organisms cultured by HDI

	High HDI (n = 15)	Middle HDI (n = 9)	Low HDI (n = 25)	Total (n = 49)
Coliform	5 (33·3)	2 (22·2)	16 (64·0)	23 (46·9)
Staphylococcus Aureus	2 (13·3)	4 (44·4)	5 (20·0)	11 (22·4)
Other organism	3 (20·0)	1 (11·1)	1 (4·0)	5 (10·2)
Anaerobe	1 (6·7)	1 (11·1)	0 (0·0)	2 (4·1)
Anaerobe+Coliform	2 (13·3)	0 (0·0)	0 (0·0)	2 (4·1)
Pseudomonas	1 (6·7)	0 (0·0)	1 (4·0)	2 (4·1)
Anaerobe+Coliform+Pseudomonas	0 (0·0)	1 (11·1)	0 (0·0)	1 (2·0)
Anaerobe+Coliform+Staphylococcus Aureus	0 (0·0)	0 (0·0)	1 (4·0)	1 (2·0)
Coliform+Staphylococcus Aureus	0 (0·0)	0 (0·0)	1 (4·0)	1 (2·0)
Streptococci Species	1 (6·7)	0 (0·0)	0 (0·0)	1 (2·0)

HDI – Human Development Index. Data are presented as n (%).
